# UV Spectrophotometric Simultaneous Determination of Paracetamol and Ibuprofen in Combined Tablets by Derivative and Wavelet Transforms

**DOI:** 10.1155/2014/313609

**Published:** 2014-02-19

**Authors:** Vu Dang Hoang, Dong Thi Ha Ly, Nguyen Huu Tho, Hue Minh Thi Nguyen

**Affiliations:** ^1^Department of Analytical Chemistry and Toxicology, Hanoi University of Pharmacy, 13-15 Le Thanh Tong, Hanoi, Vietnam; ^2^College of Education-Gia Lai, 126 Le Thanh Ton, Pleiku, Gia Lai, Vietnam; ^3^Center for Computational Science and Faculty of Chemistry, Hanoi National University of Education, Hanoi, Vietnam

## Abstract

The application of first-order derivative and wavelet transforms to UV spectra and ratio spectra was proposed for the simultaneous determination of ibuprofen and paracetamol in their combined tablets. A new hybrid approach on the combined use of first-order derivative and wavelet transforms to spectra was also discussed. In this application, DWT (sym6 and haar), CWT (mexh), and FWT were optimized to give the highest spectral recoveries. Calibration graphs in the linear concentration ranges of ibuprofen (12–32 mg/L) and paracetamol (20–40 mg/L) were obtained by measuring the amplitudes of the transformed signals. Our proposed spectrophotometric methods were statistically compared to HPLC in terms of precision and accuracy.

## 1. Introduction

**Figure 1:**
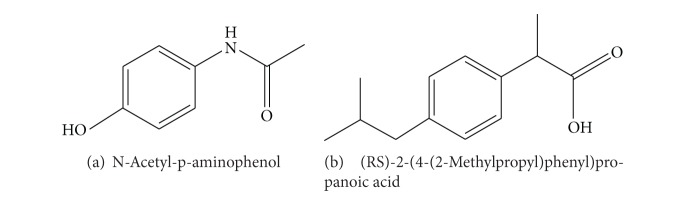
Chemical structure of paracetamol (a) and ibuprofen (b).

Fever is a common symptom, which consistently causes parental and professional phobia leading to the widespread use of antipyretic medications [1, 2]. Paracetamol (Figure 1(a)) and ibuprofen (Figure 1(b)) are commonly used over-the-counter (OTC) antipyretic drugs, especially for pediatric treatment [3–5], because they are on their own effective, safe, and relatively inexpensive.

It is postulated that paracetamol blocks prostaglandin synthesis in the hypothalamus via inhibition of cyclo-oxygenase-3 (COX-3), a splice variant of COX-1 that is mainly found in the brain and spinal cord. In contrast, ibuprofen's mechanism of action is to block the production of prostaglandins by peripherally inhibiting COX-2. These drugs could be served as a maker of soft tissue infection as persistent high fever is observed in patients receiving ibuprofen or paracetamol after varicella [6].

Despite the lack of official recommendations from guidelines in the United States and United Kingdom, it is believed that combination treatment with ibuprofen and paracetamol is beneficial over either agent alone for sustained fever reduction in children older than 6 months [7]. This combination therapy was favored for achieving an afebrile state and sustaining it [8–11]. It was found that ibuprofen is as or more efficacious than paracetamol for the treatment of pain and fever in adult and pediatric populations and is equally safe [12]. On the other hand, the combination was only slightly better on a few outcomes than ibuprofen alone; but there was a possible risk of excess dosing with the combination [13]. According to Purssell's systematic review, there is little evidence of any benefit or harm from the combined treatment compared with the use of each drug alone [14]. In the absence of such data, combining paracetamol and ibuprofen for fever in children was still questioned [15–17]. Clinically, this combination was also studied for pain relief [18–21] or postdose symptom alleviation [22].

In the field of applied UV-Vis spectroscopy, the analysis of pharmaceutical multicomponent mixtures without prior separation step is always a difficult task due to overlapping spectral peaks. Although the derivative approach still continues to be a promising tool to solve this problem [23, 24], it may have drawbacks in some cases such as (i) the higher order differentiation process diminishes peak amplitude as well as signal-to-noise ratio; (ii) the finding of zero-crossing points is very difficult and ratio spectra derivative working wavelength is undetermined. These drawbacks can be eliminated by applying wavelet transform approach to the original absorption spectra. The wavelet transform can be regarded as mathematical functions that cut up data into different frequency components and then study each component with a resolution matched to its scale. It is a powerful tool for signal processing in many branches of science and engineering. In the last decade, its applications in analytical chemistry, for example, data reduction [25], denoising [26], and baseline correction [27], have been recorded. The wavelet-based resolution of multicomponent pharmaceutical mixtures has also been exploited, for which quaternary mixtures containing paracetamol are an example [28].

In the literature, the determination of paracetamol and ibuprofen could be simultaneously performed by HPLC, CE, and HPTLC [29–32]. Their spectrophotometric determination was also studied by applying chemometrics and derivative approach [33–35]. It is noteworthy that differentiation and smoothing algorithms for UV derivative spectrophotometry in these studies were not clearly indicated, whereas they always play an important role in determining the sensitivity and accuracy of derivative techniques.

The aim of this study was to develop derivative- and wavelet-based UV spectrophotometric methods for the simultaneous determination of paracetamol and ibuprofen in their combined tablets using HPLC as a reference method. This study, in particular, emphasized on exploiting the advantages of wavelet transform over differentiation algorithms (i.e., continuous, discrete, and fractional wavelet transform) as well as correcting the shortcomings in the above-mentioned UV derivative spectrophotometry studies.

## 2. Experimental

### 2.1. Apparatus and Software

Absorption spectra were registered and treated by using a UNICAM UV 300 double beam spectrophotometer (Thermo Spectronic, USA) with a fixed slit width (1.5 nm) connected to an IBM computer loaded with Thermo Spectronic VISION32 software and 1-cm quartz cells. The zero-order spectra were recorded in the wavelength range of 200–325 nm against a blank (phosphate buffer pH 7.2) at Intelliscan mode to enhance the signal-to-noise ratio of absorbance peaks without extended scan duration with a Δ*λ* = 0.1 nm (i.e., 30–120 nm/min). For derivative approach, the spectra were differentiated and smoothed by using Savitzky-Golay filter. For wavelet approach, the data treatment was done using MATLAB R2013a software (The MathWorks, Natick, MA, USA). FWT calculations were performed in MATLAB with its code of FWT performed by Unser and Blu. 

High performance liquid chromatogram (HPLC) analysis was performed on an Agilent 1100 Series Diode-Array-Detector chromatograph (Agilent Technologies, USA) at ambient temperature. An Eclipse XDB-C18 (3 × 150 mm, 3.5 *μ*m) column was used. All solutions were filtered through a 0.45 *μ*m membrane filter before injection into the chromatograph. All solvents were filtered through a 0.45 *μ*m Millipore filter and degassed in an ultrasonic bath.

### 2.2. Reagents and Standard Solutions

Paracetamol, PA (99.5%), and ibuprofen, IB (100.0%), were kindly provided by the National Institute of Drug Quality Control (Vietnam). Deionized doubly distilled water was used throughout. All reagents were of an analytical grade. Stock solutions of PA and IB (500 mg/L) were freshly made in phosphate buffer pH 7.2. A concentration set of standard solutions were prepared in 25 mL calibrated flasks by using the same stock solutions.

### 2.3. Sample Solution

Three commercial formulations containing paracetamol 325 mg + ibuprofen 200 mg per tablet were studied, that is, Alaxan (United Pharma, Vietnam), Dibulaxan (Danapha Pharmaceutical Joint Stock Company, Vietnam), and Febro (OPV Pharmaceutical Joint Stock Company, Vietnam). For each formulation, twenty tablets were finely pulverized in a mortar. A quantity equivalent to one tablet was accurately weighed and dissolved in about 50 mL of phosphate buffer pH 7.2 in a 100 mL volumetric flask by sonication for 20 min and subsequently diluted to the mark with the same solvent. Appropriate dilution was then made in a 25 mL volumetric flask to obtain the test solution ca. 32.5 mg/L paracetamol + 20 mg/L ibuprofen.

## 3. Theoretical Background

The theoretical background of derivative transform and smoothing of signals using Savitzky-Golay method [36] as well as fundamentals of CWT, DWT [37], and FWT [38] are briefly described as follows.

### 3.1. Derivative Transform Approach

#### 3.1.1. Savitzky-Golay Method

This method determines a derivative spectrum by moving a spectral window comprising 2*n* + 1 measurement points over an absorbance spectrum. Then a polynomial of order m is fitted to the measurement points inside the spectral window as follows:

(1) $$\begin{array}{c} P\left(\lambda \right)=a_{0}+a_{1}\lambda +\;a_{2}\lambda^{2}+\cdots +a_{m}\lambda^{m}. \end{array}$$

This fit polynomial introduces smoothing, which is dependent on the user selectable parameters *n* and *m*. From the resulting fit parameters *a*_0_,…, *a*_*m*_, the derivatives at the window center *λ*_0_ can be derived easily as follows:

(2) $$\begin{array}{c} {\frac{dP}{d\lambda }|}_{\lambda_{0}=0}=a_{1}+2a_{2}\lambda +\cdots +ma_{m}\lambda^{m-1}=a_{1},\\ {\frac{d^{2}P}{d\lambda^{2}}|}_{\lambda_{0}=0}=2a_{2}+\cdots +m\left(m-1\right)a_{m}\lambda^{m-2}=2a_{2},\\ {\frac{d^{3}P}{d\lambda^{3}}|}_{\lambda_{0}=0}=6a_{3}+\cdots +m\left(m-1\right)\left(m-2\right)a_{m}\lambda^{m-3}=6a_{3}. \end{array}$$

Once the derivatives are determined at *λ*_0_, the window is moved one measurement point to the right followed by a polynomial fit inside this new window until it reaches the end of the spectrum.

### 3.2. Wavelet Transform Approach

#### 3.2.1. Continuous Wavelet Transform (CWT)

Given a time-varying signal *f*(*t*), the wavelet transforms consist of computing coefficients, which are inner products of the signal and a family of wavelets. In a continuous wavelet transform, the wavelet corresponding to scale *a* and time location *b* can be written in terms of the mother wavelet as follows:

(3) $$\begin{array}{c} \psi_{a,b}\left(t\right)=\;\frac{1}{\sqrt{\left|a\right|}}\;\psi \left(\frac{t\;-\;b}{a}\right)\;\text{with}\;a,b\in R,a\;\neq \;0.\; \end{array}$$

The continuous wavelet transform (CWT) of*f*(*t*) is given by

(4) $$\begin{array}{c} W_{f}\left(a,b\right)=\overset{\infty }{\underset{-\infty }{\int }}f\left(t\right)\psi_{a,b}\left(t\right)dt. \end{array}$$

The inversion back to time domain is given by

(5) $$\begin{array}{c} f\left(t\right)=\frac{1}{C_{\psi }}\overset{\infty }{\underset{-\infty }{\int }}\frac{1}{a^{2}}\left[\overset{\infty }{\underset{-\infty }{\int }}W_{f}\left(a,b\right)\psi_{a,b}\left(t\right)db\right]da. \end{array}$$

When a continuous wavelet transform is evaluated, the mother wavelet is scaled and translated to every possible value of *a* and *b*. Accordingly, at each location (translation) of the wavelet, information is obtained about the local contribution of each frequency (scaling) to the entire signal.

#### 3.2.2. Discrete Wavelet Transform (DWT)

When the discrete wavelet transform is used to analyze digitized signals, the scaling and the translation of the mother wavelet will be

(6) $$\begin{array}{c} \psi_{m,n}\left(t\right)=\frac{1}{a_{0}^{m/2}}\psi \left(\frac{t\;-\;nb_{0}}{a_{0}^{m}}\right). \end{array}$$

The discrete wavelet transform is then written as

(7) $$\begin{array}{c} W_{f}\left(m,n\right)=\overset{\infty }{\underset{-\infty }{\int }}f\left(t\right)\psi_{m,n}\left(t\right)dt. \end{array}$$

Usually, *a*_0_ = 2 and *b*_0_ = 1 values are chosen. The wavelet transform calculated is called dyadic when *a*_0_ = 2.

#### 3.2.3. Fractional Wavelet Transform (FWT)

*B-Spline*. A B-spline is defined as a generalization of the Bezier curve. Let a vector known as the knot be defined as   *T* = {*t*_0_, *t*_1_,…, *t*_*m*_}, where *T* is a nondecreasing sequence with *t*_*i*_ ∈[0,1], and the control points are defined as *P*_0_, *P*_*n*_. Degree is defined as *p* = *m* − *n* − 1. The knots *t*_*p*+1_,…, *t*_*m*−*p*−1_ are called internal knots. If the basis functional is defined as

(8) $$\begin{array}{c} N_{i,0}\left(t\right)=\{\begin{array}{cc} 1, & \text{if}\;t_{i}\;\leq \;t<t_{i+1},\;t_{i}<t_{i+1},\\ 0, & \text{otherwise}, \end{array}\\ N_{i,p}\left(t\right)=\frac{t\;-\;t_{i}}{t_{i+p}-\;t_{i}}N_{i,p-1}\left(t\right)+\frac{t_{i+p+1}-t}{t_{i+p+1}-t_{i+1}}N_{i+1,p-1}\left(t\right), \end{array}$$

then the curve defined by

(9) $$\begin{array}{c} C\left(t\right)=\overset{n}{\underset{i=0}{\sum }}P_{i}N_{i,p}\left(t\right) \end{array}$$

is a B-spline.

*Fractional B-Spline.* The fractional B-spline is defined as

(10) $$\begin{array}{c} \beta_{+}^{\alpha }\left(x\right)=\;\frac{\Delta_{+}^{\alpha +1}x_{+}^{\alpha }}{\Gamma \left(\alpha +1\right)}=\frac{\sum_{k=0}^{+\infty }{\left(-1\right)}^{k}\left(\begin{array}{c} \alpha +1\\ k \end{array}\right){\left(x-k\right)}_{+}^{\alpha }}{\Gamma \left(\alpha +1\right)}, \end{array}$$

where Euler's gamma function is defined as follows:

(11) $$\begin{array}{c} \Gamma \left(\alpha +1\right)=\overset{+\infty }{\underset{0}{\int }}x^{\alpha }e^{-x}dx,\\ {\left(x-k\right)}_{+}^{\alpha }=\max{\left(x-k,0\right)}^{\alpha }. \end{array}$$

The forward fractional finite difference operator of order  *α*  is defined as

(12) $$\begin{array}{c} \Delta_{+}^{\alpha }f\left(x\right)=\overset{+\infty }{\underset{0}{\sum }}{\left(-1\right)}^{k}\left(\begin{array}{c} \alpha \\ k \end{array}\right)\;f\left(x-k\right), \end{array}$$

where

(13) $$\begin{array}{c} \left(\begin{array}{c} \alpha \\ k \end{array}\right)=\;\frac{\Gamma \left(\alpha +1\right)}{\Gamma \left(k+1\right)\Gamma \left(\alpha -k+1\right)}. \end{array}$$

The above-defined B-splines fulfill the convolution property as follows:

(14) $$\begin{array}{c} \beta_{+}^{\alpha_{1}}\;\times \;\beta_{+}^{\alpha_{2}}=\beta_{+}^{\alpha_{1}+\alpha_{2}}. \end{array}$$

The centered fractional B-splines of degree *α* are given by

(15) $$\begin{array}{c} \beta_{*}^{\alpha }\left(x\right)=\frac{1}{\Gamma \left(\alpha +1\right)}\underset{k\in Z}{\sum }{\left(-1\right)}^{k}\left|\begin{array}{c} \alpha +1\\ k \end{array}\right|\;{\left|x-k\right|}_{*}^{\alpha }, \end{array}$$

where |*x*|_∗_^*α*^ has the following form:

(16) $$\begin{array}{c} {\left|x\right|}_{*}^{\alpha }=\{\begin{array}{cc} \frac{{\left|x\right|}^{\alpha }}{-2\sin\left(\left(\pi /2\right)\alpha \right)}, & \alpha \;\text{not}\;\text{even},\\ \frac{x^{2n}\log x}{{\left(-1\right)}^{1+n}\pi }, & \alpha \;\text{even}. \end{array} \end{array}$$

*Fractional B-Spline Wavelets.* The fractional B-spline wavelets are defined as follows:

(17) ψ+α(x2)=∑k∈Z(−1)k2α∑1∈Z(α+11) ×  β∗2α+1(1+k−1)β+α(x−k).

The fractional splines wavelets obey

(18) $$\begin{array}{c} \overset{+\infty }{\underset{-\infty }{\int }}x^{n}\psi_{+}^{\alpha }\left(x\right)dx=0, \end{array}$$

and the Fourier transform fulfills the following relations:

(19) $$\begin{array}{c} {\hat{\psi }}_{*}^{\alpha }\left(\varpi \right)=\;C{\left(j\varpi \right)}^{\alpha +1},\;\;\text{as}\;\omega ⟶0,\\ {\hat{\psi }}_{*}^{\alpha }\left(\varpi \right)=C{\left(j\varpi \right)}^{\alpha +1},\;\text{as}\;\omega ⟶0. \end{array}$$

Here, ${\hat{\psi }}_{*}^{\alpha }(\varpi )$ is symmetric. The fractional spline wavelets behave like fractional derivative operators as indicated by the last formulas.

## 4. Results and Discussion

**Figure 2:**
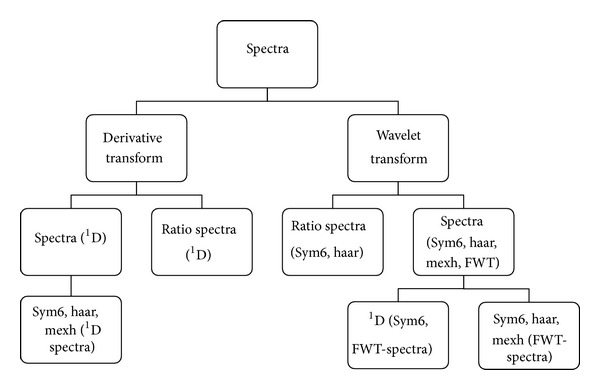
Derivative and wavelet transforms for the simultaneous determination of IB and PA in binary mixtures.

**Figure 3:**
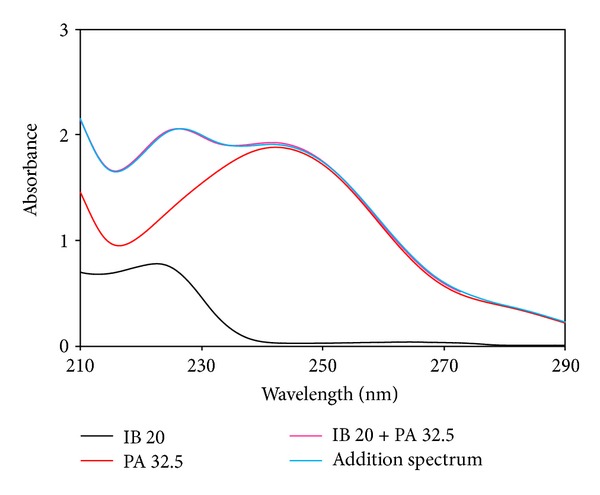
Spectra of IB 20 mg/L, PA 32.5 mg/L, and their corresponding mixture and absorbance addition.

For spectrophotometric measurements, phosphate buffer pH 7.2, a medium successfully studied for the dissolution test of PA and IB combined tablets [39], was chosen to solubilize both drugs.  Figure 3 shows the zero-order UV absorption spectra after being smoothed by Savitzky-Golay algorithm (Order: 3; number of coefficients: 125). It is clear that (i) the additivity of absorbances was obeyed for the mixture of IB 20 mg/L + PA 32.5 mg/L and (ii) the determination of IB in the mixture was impossible because the spectrum of PA 32.5 mg/L completely covered the spectrum of IB 20 mg/L over the range 210–290 nm. In order to determine simultaneously IB and PA in binary mixtures, their overlapping spectra were resolved using derivative and wavelet transforms as graphically depicted in Figure 2.

In principle, derivative and wavelet transforms could be applied to spectra or ratio spectra. While finding zero-crossing or crossing points is crucial to the transformed spectra, the applicability of the transformed ratio spectra depends on finding a point or a region over which the coincidence of derivative or wavelet signals is observed for the ratio spectra of a compound and its corresponding mixture.

### 4.1. Method Development

#### 4.1.1. Derivative Transform

**Figure 4:**
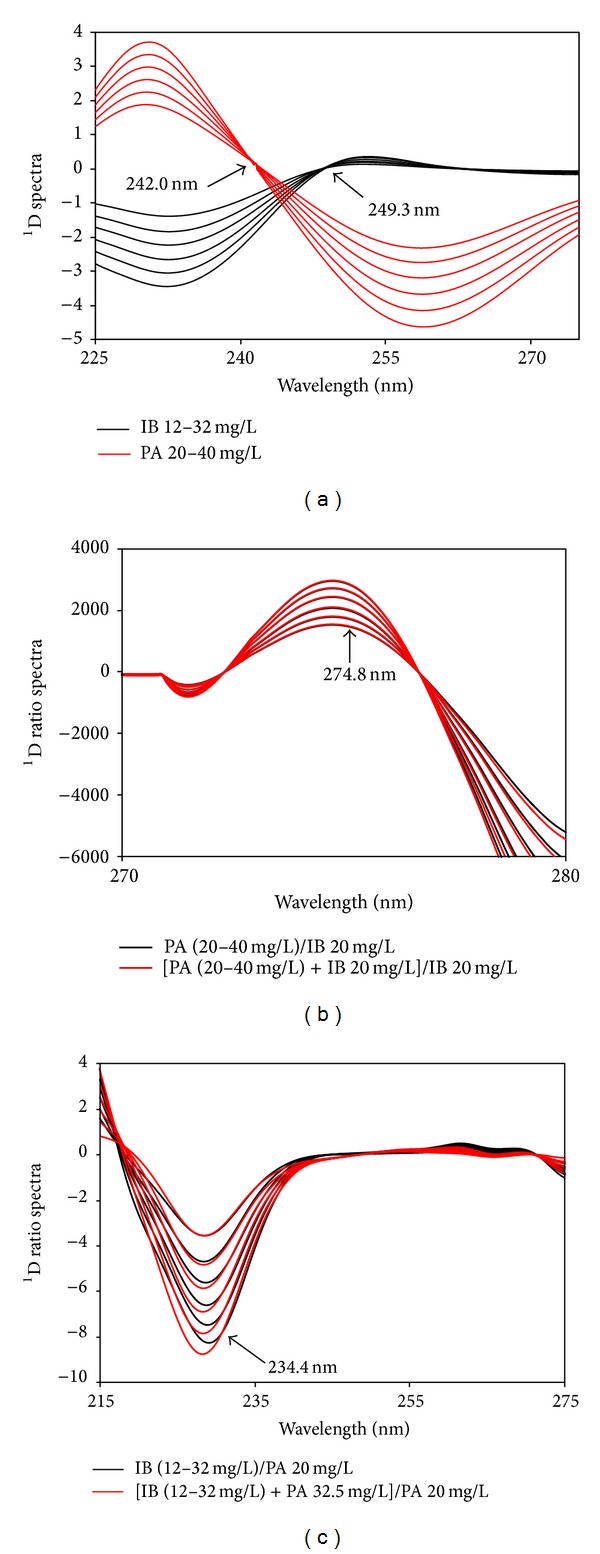
First-order derivatives of spectra (a) and first-order derivatives of ratio spectra (b) and (c).

Figure 4(a) displays the first derivative spectra of these pure drugs after their original spectra being differentiated (Order: 5; number of coefficients: 9) and smoothed (Order: 3; number of coefficients: 501) by Savitzky-Golay algorithm, which reveals that there existed zero-crossing points at 249.3 and 242.0 nm for IB and PA, respectively. Both wavelengths were subsequently chosen for the simultaneous determination of PA and IB due to their derivative amplitudes proportional to the concentration ranges studied of PA (20–40 mg/L) and IB (12–32 mg/L).

Figures 4(b) and 4(c) present the first-order derivatives of ratio spectra after ratio spectra being differentiated (Order: 5; number of coefficients: 9) and smoothed (Order: 3; number of coefficients: 125) by Savitzky-Golay algorithm. To optimize this technique, the influence of divisor standard concentration was investigated with the concentration ranges for Lambert-Beer's law compliance. A standard spectrum of 20 mg/L was considered suitable for the determination of both drugs. The determination of each component was based on the proportionality of its concentrations to relevant first-order derivative amplitudes at a suitable wavelength. The two points, 274.8 and 234.4 nm, at which the highest amplitude and coincidence of derivative signals were seen with an error less than 3%, were selected as the working wavelengths for analyzing PA and IB, respectively. The fact that our data are different from previously published works on spectrophotometric simultaneous determination of IB and PA in their mixture [34, 35] could be attributed to the difference in differentiating and smoothing manner, solvent, and equipment used. Nevertheless, our experimental setup seems to be better than these studies when referring to (i) the use of water, an ecofriendly solvent, rather than methanol and (ii) higher amplitudes of derivative signals obtained for the same concentration range.

#### 4.1.2. Wavelet Transform

In practice, wavelet transform of spectra and ratio spectra for the determination of IB and PA was carried out by transferring spectra data vectors into the wavelet domain and then applying wavelet transform (CWT, DWT, and FWT) to the signal data in the wavelet domain.

**Figure 5:**
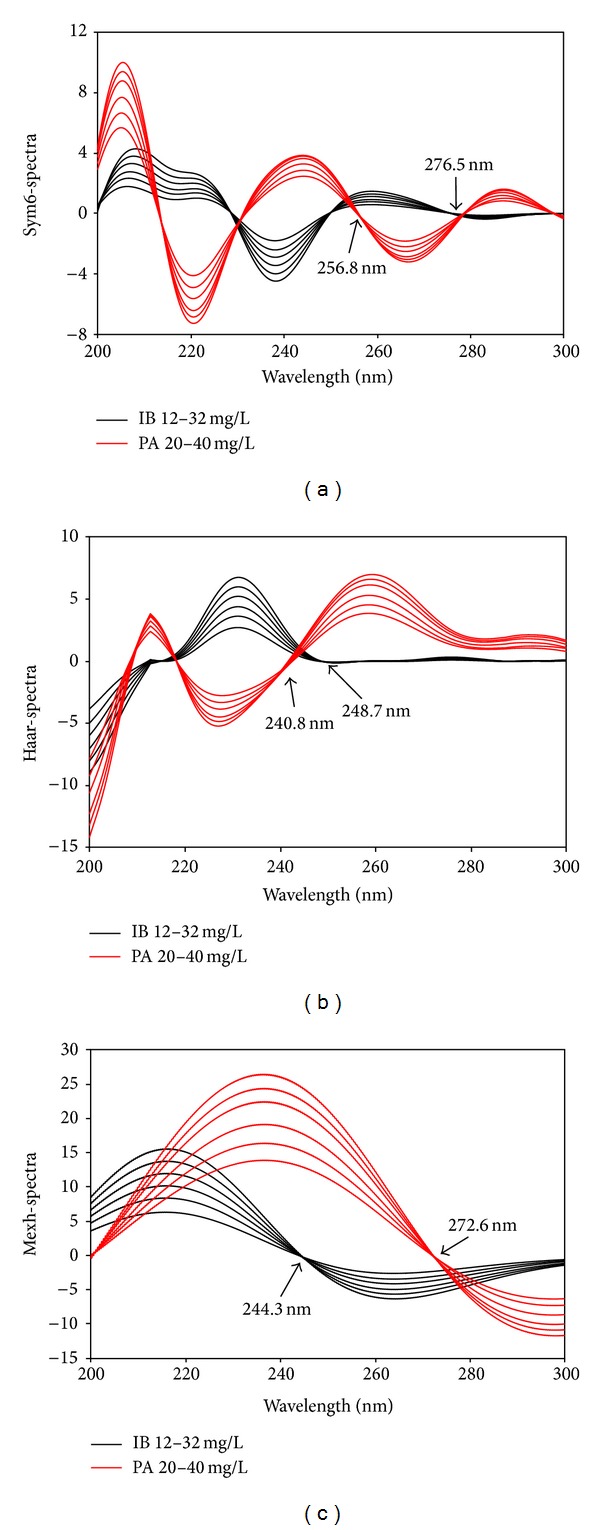
Wavelet transform of spectra using sym6 (a), haar (b), and mexh (c).

**Figure 6:**
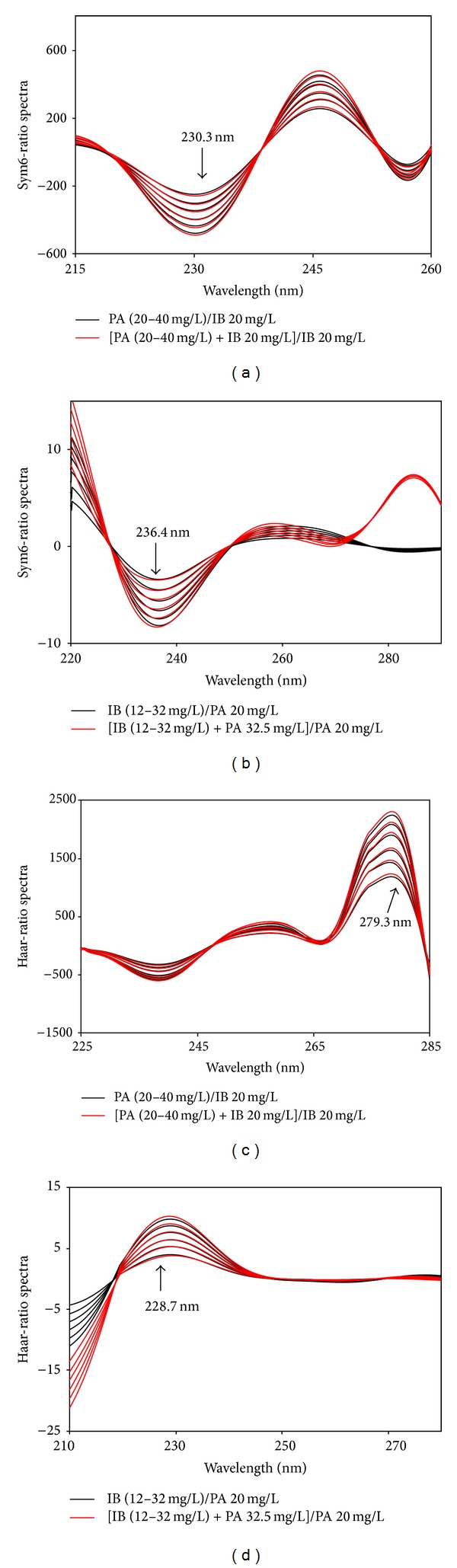
Wavelet transform of ratio spectra using sym6 (a) and (b) and haar (c) and (d).

For the optimization of the wavelet analysis, various wavelet transform methods at different dilation parameters (*a*) were tested to identify wavelet transform families in order to provide the best spectral recovery values. In the above test, Sym6, Haar, and Mexh were found to be appropriate for the transformation of spectral signals of the two compounds and their mixtures. On the other hand, several dilation parameters (*a*) with frequency (*f*) for these CWT and DWT approaches were tested to find the optimal signal processing parameters. For this, *a* = 256 with *f* = 0.182 (sym6), *f* = 0.249 (haar), and *f* = 0.063 (mexh) were determined. The application of these families to resolve spectra and ratio spectra is displayed in Figures 5 and 6.

#### 4.1.3. Derivative-Wavelet Transforms Combined

**Figure 7:**
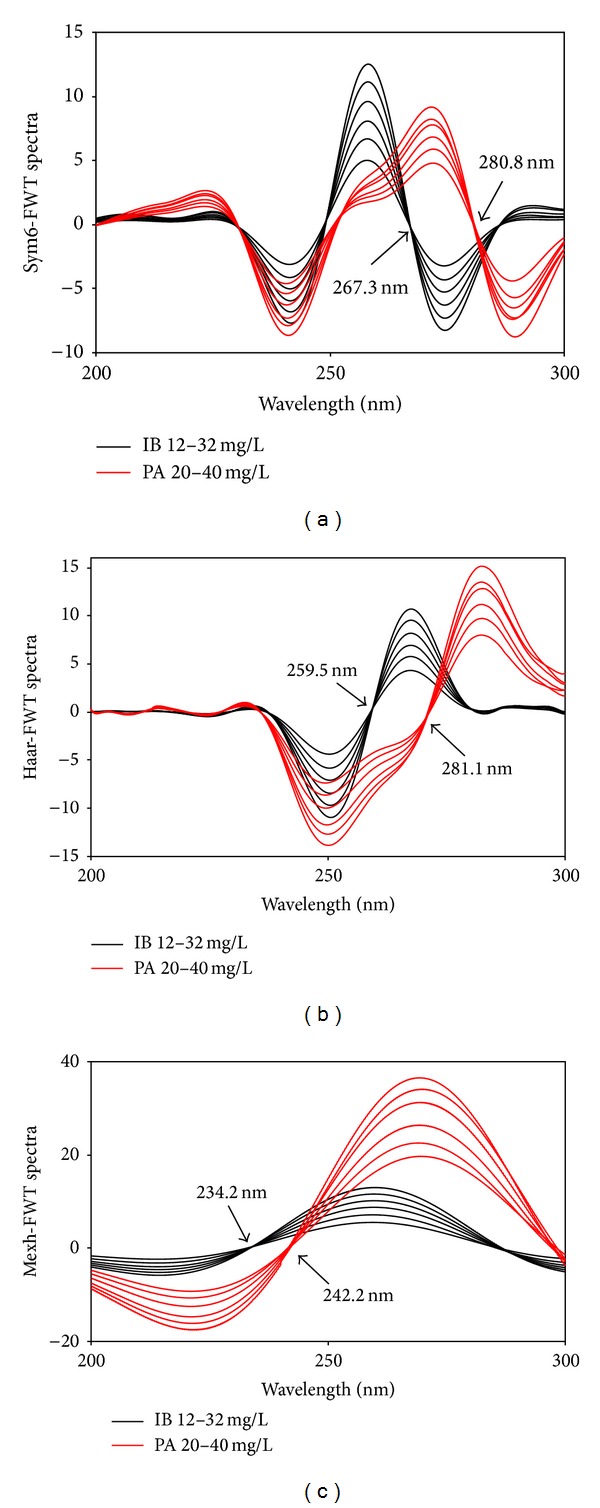
Wavelet transform (sym6 (a), haar (b), and mexh (c)) of FWT-modified spectra.

FWT is a new promising method in signal and image analysis, which offers the functions of data compression and denoising to effectively extract the important form of complex original spectra. It is noticeable that the selected columns among the whole FWT coefficients contain low frequency information in high scales; that is, the absorption spectrum is smooth and possesses high amplitude. In this study, FWT signal analysis approach was applied to the zero-order absorption spectra in the wavelength range of 200.0–302.3 nm (i.e., 1024 points). Several parameters *α* and depths of the decomposition (*J*) were tested for optimizing the fractional signal processing. *α* = − 0.3 and *J* = 1 were found to be the optimal ones. The type of B-splines was considered to be causal orthonormal. After that, the FWT spectra were subjected to further wavelet transform (sym6, haar, and mexh) to find zero-crossing points for the simultaneous determination of IB and PA in their mixtures (Figures 7(a), 7(b), and 7(c)).

**Figure 8:**
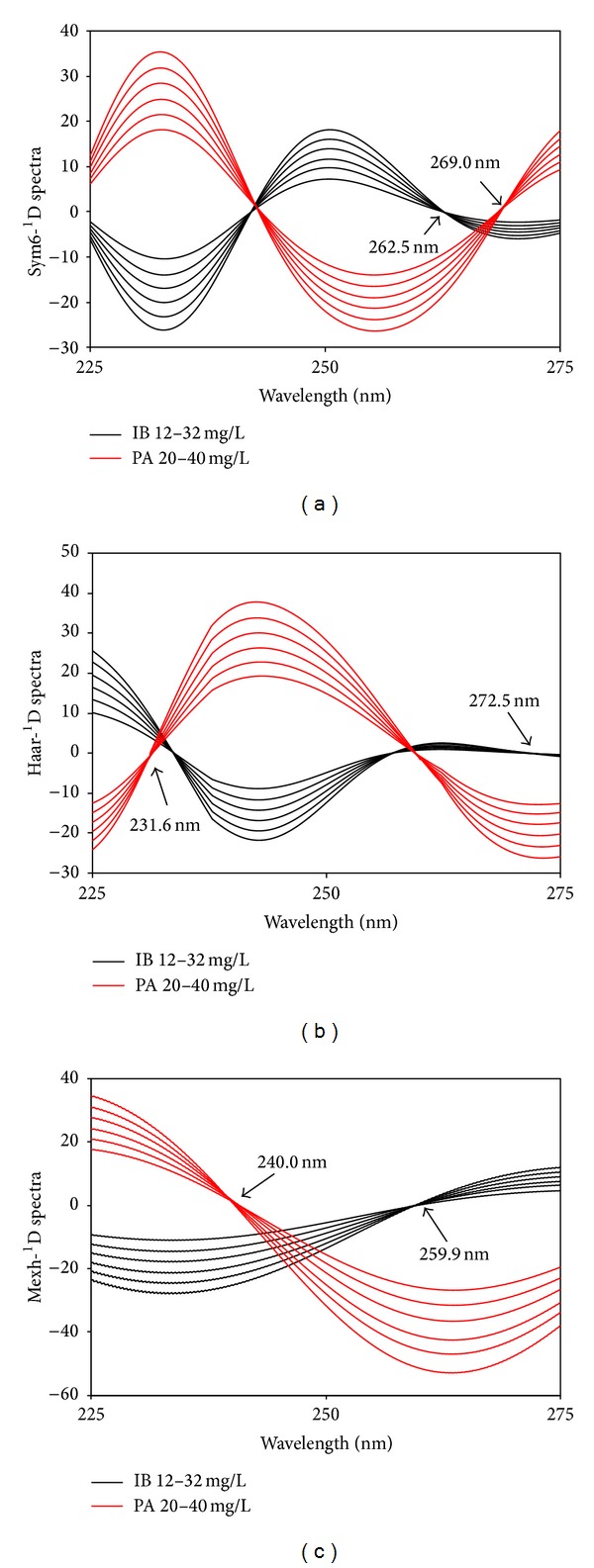
Wavelet transform of first-order derivative spectra using sym6 (a), haar (b), and mexh (c).

**Figure 9:**
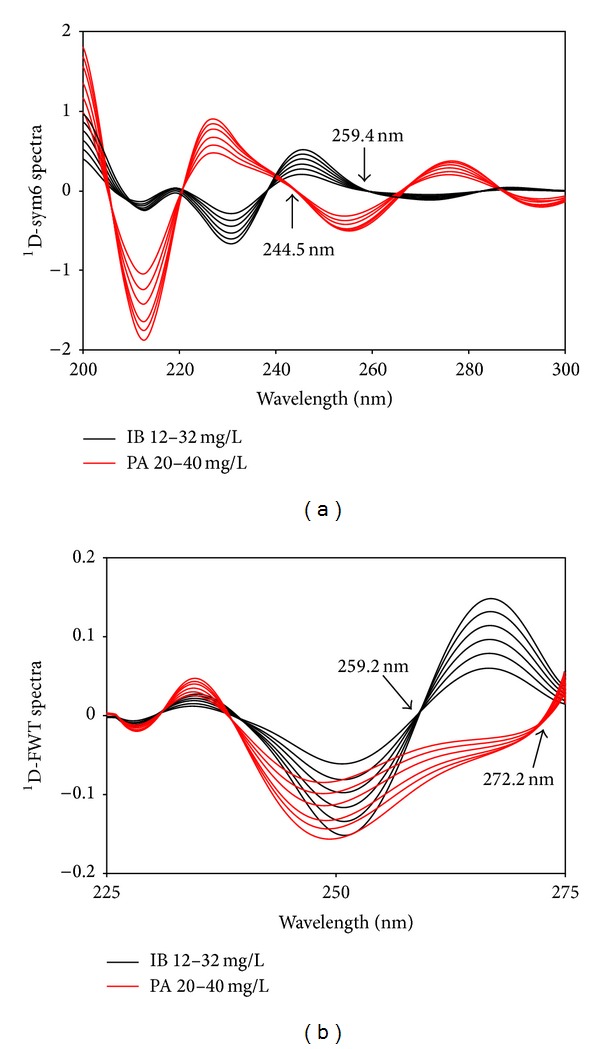
Derivative transform of wavelet transformed spectra by sym6 (a) and FWT (b).

In another development, the combination of derivative and wavelet transforms was performed in an effort to increase the number of zero-crossing points as well as to obtain a higher sensitivity and selectivity as compared to the original derivative or wavelet spectra. This approach was successfully done with wavelet transform (sym6, haar, and mexh) of the first-order derivative spectra (Figures 8(a), 8(b), and 8(c)) and the first-order derivative transform of the wavelet transformed spectra (Figures 9(a) and 9(b)).

#### 4.1.4. High Performance Liquid Chromatography Analysis

**Figure 10:**
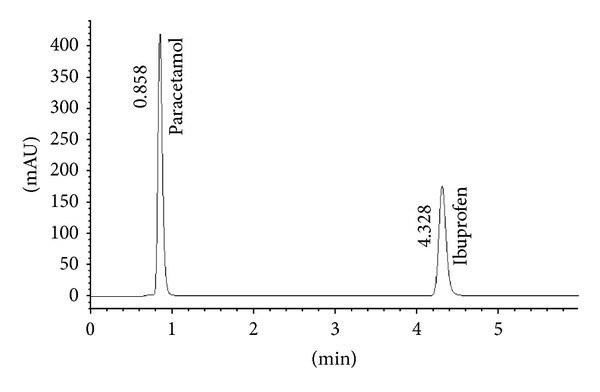
Typical liquid chromatogram of a binary mixture of IB 20 mg/L and PA 32.5 mg/L.

The reversed-phase HPLC for the analysis of binary mixtures containing IB and PA was developed as a reference method. The optimization of HPLC analysis was as follows. IB and PA were chromatographically analyzed by isocratic elution with a flow rate of 0.8 mL/min. The mobile phase composition was acetonitrile-phosphoric acid 0.1% (55 : 45, v/v). Injection volume was 20 *μ*L and detection wavelength was 221.0 nm for both compounds. Under our chromatographic conditions, the retention time was found to be 0.86 and 4.33 min for PA and IB, respectively (Figure 10). The chromatographic parameters such as resolution (Rs = 15.4), peak asymmetry (AF = 0.8), and plate number (ca. 1000/15 cm) were satisfactory for both compounds obviously confirming the suitability of our HPLC method.

### 4.2. Method Validation

The validity of the proposed HPLC method was assessed by accuracy, precision, and linearity. For studying the accuracy, known quantities of IB and PA (i.e., 10–20% nominal content of their combined tablets) were added to each predetermined pharmaceutical formulation. The amount of analyte recovered was expressed as average percent recovery with the upper and lower limits of standard deviation. The average percent recoveries obtained were 99.5 ± 0.9 and 100.3 ± 1.0% for PA and IB, respectively, indicating the method's good accuracy and no marked interference by common excipients in the tablets studied. The within-run precision (repeatability) of HPLC was evaluated by analyzing six replicates of the same formulation a day. The low RSD values (<2.0%) indicate the method's good precision. By analogy, the proposed spectrophotometric methods also showed good precision (RSD < 2.0%). The calibration graphs for HPLC and UV spectrophotometric determination with the linear concentration ranges of IB (12–32 mg/L) and PA (20–40 mg/L) are summarized in Table 1.

The proposed techniques were successfully applied to the simultaneous determination of IB and PA in their combined tablets. The spectrophotometric results were statistically compared with those obtained by HPLC (Table 2). It is seen that, at 95% confidence level, there was no significant difference between the accuracy (evaluated by ANOVA test, calculated *F* value < tabulated *F* value) and precision (evaluated by Bartlett test, calculated *χ*2 value < tabulated *χ*2 value) among all the proposed methods (Table 3).

## 5. Conclusion

UV spectrophotometric methods based on first-order derivative transform, CWT (mexh), DWT (sym6 and haar), and FWT of spectra and ratio spectra were developed for the spectra resolution of IB and PA in their binary mixtures without prior separation step. In particular, a new hybrid approach on the combined use of derivative and wavelet transform was also suggested. The application of wavelet transform to UV spectra showed some advantages over derivative spectrophotometry such as higher peak intensity obtained, additional smooth function, and scaling factor process eliminated. All the proposed spectrophotometric methods were simple and statistically compared to liquid chromatographic data in terms of precision and accuracy. It offers possible interchangeability between UV spectrophotometric methods and HPLC for the simultaneous determination of IB and PA in their combined tablets.

## Figures and Tables

**Table 1:** Statistical analysis of calibration graphs of the proposed HPLC and spectrophotometric methods (*n* = 6), IB (12–32 mg/L) and PA (20–40 mg/L).

Method	Compound	Wavelength (nm)	*a*	*b*	*S*_*a*_	*S*_*b*_	*S*_*y*·*x*_	*R* ^2^
HPLC	IB	221.0	57.684	−86.417	0.2861	6.5906	4.7873	0.9999
	PA	221.0	48.886	−25.095	0.2636	8.2531	4.2943	0.9998

Derivative transform								
^ 1^D spectra	IB	242.0	−0.0521	−0.0721	0.0005	0.0114	0.0083	0.9996
	PA	249.3	−0.0731	0.0689	0.0019	0.0608	0.0331	0.9970
^ 1^D ratio spectra	IB	230.4	−0.2385	−0.5009	0.0056	0.1310	0.0951	0.9977
	PA	274.8	72.666	89.283	1.3424	41.421	22.552	0.9986

Wavelet transform								
Sym6-spectra	IB	256.8	0.0445	−0.5066	0.0005	0.0116	0.0084	0.9997
	PA	276.5	−0.0248	0.0735	0.0005	0.0161	0.0087	0.9982
Haar-spectra	IB	240.8	0.0820	−0.6405	0.0013	0.0301	0.0218	0.9989
	PA	248.7	0.0964	0.3651	0.0018	0.0584	0.0318	0.9984
Mexh-spectra	IB	272.6	−0.1710	−0.5047	0.0051	0.1196	0.0869	0.9963
	PA	244.3	0.6333	0.9753	0.0149	0.4610	0.2510	0.9977
Sym6-ratio spectra	IB	236.4	−0.2416	−0.6292	0.0033	0.0764	0.0555	0.9992
	PA	230.3	−10.291	−58.576	0.3182	9.8194	5.3461	0.9961
Haar-ratio spectra	IB	228.7	0.3189	0.0935	0.0040	0.0922	0.0670	0.9993
	PA	279.3	53.276	155.16	0.7739	23.879	13.001	0.9991

Derivative-Wavelet transforms combined								
Sym6-^1^D spectra	IB	268.3	−0.1691	−0.1659	0.0013	0.0310	0.0225	0.9997
	PA	262.5	−0.4605	−0.0536	0.0112	0.3483	0.1896	0.9976
Haar-^1^D spectra	IB	231.6	0.2246	0.7973	0.0059	0.1365	0.0991	0.9972
	PA	272.5	−0.6666	0.6464	0.0188	0.5828	0.3173	0.9967
Mexh-^1^D spectra	IB	240.5	−0.7507	−1.0118	0.0064	0.1487	0.1080	0.9997
	PA	259.9	−1.2642	−0.3523	0.0191	0.5922	0.3224	0.9990
^ 1^D-Sym6 spectra	IB	244.5	0.0160	−0.0071	0.0002	0.0060	0.0044	0.9989
	PA	259.4	−0.0090	−0.0904	0.0004	0.0124	0.0068	0.9921
^ 1^D-FWT spectra	IB	272.2	0.0025	0.0007	0.0001	0.0003	0.0002	0.9998
	PA	259.2	−0.0024	0.0074	0.0001	0.0016	0.0008	0.9981
Sym6-FWT spectra	IB	280.8	−0.1376	0.0033	0.0029	0.0680	0.0494	0.9981
	PA	267.3	0.2029	0.1088	0.0044	0.1371	0.0746	0.9980
Haar-FWT spectra	IB	270.7	0.2745	0.4080	0.0034	0.0799	0.0580	0.9993
	PA	259.5	−0.2289	0.3976	0.0084	0.2595	0.1413	0.9946
Mexh-FWT spectra	IB	242.2	0.1589	0.7507	0.0057	0.1332	0.0967	0.9947
	PA	234.2	−0.2808	0.1765	0.0112	0.3469	0.1888	0.9936

*Y* = *aC* + *b*, where *C* is the concentration in mg/L and *Y* in signal's amplitude units (for spectrophotometric methods) or mAU × sec (for HPLC).

*a*: slope; *b*: intercept; *S*_*a*_: SD of the slope; *S*_*b*_: SD of the intercept; *S*_*y*·*x*_: SD of the residuals; *R* ^2^: coefficient of determination.

**Table 2:** Assay results for the determination of IB and PA in their combined tablets.

% of label claim (mean ± SD, *n* = 6)						
Method	Alaxan		Dibulaxan		Febro	
	IB	PA	IB	PA	IB	PA

HPLC	99.4 ± 0.9	99.8 ± 0.9	99.8 ± 1.3	99.4 ± 1.0	100.3 ± 1.0	99.3 ± 1.2

Derivative transform						
^ 1^D spectra	99.5 ± 0.9	100.1 ± 1.4	100.3 ± 1.7	99.6 ± 1.3	99.4 ± 0.9	99.3 ± 1.3
^ 1^D ratio spectra	99.8 ± 1.3	99.1 ± 1.1	100.2 ± 1.5	99.3 ± 0.9	99.4 ± 1.5	100.1 ± 1.4

Wavelet transform						
Sym6-spectra	100.3 ± 1.8	99.6 ± 1.3	100.3 ± 1.5	99.4 ± 1.2	100.6 ± 0.8	99.1 ± 1.0
Haar-spectra	99.3 ± 1.3	99.1 ± 1.2	100.3 ± 0.9	99.7 ± 1.7	100.5 ± 1.2	99.4 ± 0.9
Mexh-spectra	101.0 ± 1.2	100.7 ± 0.8	100.5 ± 1.8	98.8 ± 1.3	99.4 ± 0.9	99.6 ± 1.1
Sym6-ratio spectra	100.1 ± 1.1	99.1 ± 1.3	99.4 ± 0.9	100.4 ± 0.9	99.2 ± 1.1	99.8 ± 0.9
Haar-ratio spectra	99.7 ± 0.9	99.3 ± 0.6	100.1 ± 1.0	99.6 ± 1.1	100.6 ± 1.3	99.5 ± 1.1

Derivative-Wavelet transforms combined						
Sym6-^1^D spectra	100.8 ± 1.4	100.0 ± 1.0	99.7 ± 1.2	101.0 ± 1.1	99.5 ± 1.4	100.5 ± 0.9
Haar-^1^D spectra	99.5 ± 0.8	99.5 ± 1.0	100.7 ± 1.1	100.9 ± 1.3	100.7 ± 0.8	99.8 ± 0.8
Mexh-^1^D spectra	99.7 ± 1.0	99.5 ± 0.9	99.8 ± 1.3	100.2 ± 1.5	99.7 ± 0.9	99.8 ± 1.2
^ 1^D-Sym6 spectra	100.5 ± 1.1	100.5 ± 0.8	99.8 ± 1.5	99.3 ± 1.4	100.6 ± 0.8	100.0 ± 1.1
^ 1^D-FWT spectra	99.9 ± 1.4	100.4 ± 1.3	100.1 ± 1.3	99.9 ± 1.2	100.4 ± 1.1	100.4 ± 1.0
Sym6-FWT spectra	100.7 ± 1.1	100.3 ± 0.9	100.4 ± 1.1	99.7 ± 1.4	99.6 ± 0.9	100.1 ± 0.9
Haar-FWT spectra	99.8 ± 1.3	99.9 ± 1.0	99.7 ± 1.5	100.1 ± 1.0	100.4 ± 1.2	100.6 ± 1.0
Mexh-FWT spectra	100.3 ± 0.9	99.6 ± 1.4	99.5 ± 1.6	100.7 ± 0.8	100.6 ± 0.8	99.7 ± 1.3

**Table 3:** Results of ANOVA and Bartlett tests at the significance level of 5% by applying spectrophotometric and chromatographic methods to three commercial pharmaceutical formulations.

ANOVA test					
Source of variation	Compound		Between groups	Within groups	Total
Sum of squares	IB	I	25.11	110.90	136.01
		II	12.70	146.20	158.90
		III	28.56	90.00	118.56
	PA	I	24.15	93.55	117.70
		II	35.46	118.45	153.91
		III	17.99	89.90	107.89

Degree of freedom			15	80	95

Mean of squares	IB	I	1.674	1.386	
		II	0.847	1.827	
		III	1.904	1.125	
	PA	I	1.610	1.169	
		II	2.364	1.481	
		III	1.199	1.124	

Calculated *F* value	IB	I	1.207		
		II	0.464		
		III	1.692		
	PA	I	1.377		
		II	1.597		
		III	1.067		

Tabulated *F* value			1.793		

Bartlett test					
Degree of freedom			15		

Calculated *χ* ^2^ value	IB	I	6.923		
		II	6.201		
		III	6.506		
	PA	I	7.352		
		II	5.810		
		III	2.870		

Tabulated *χ* ^2^ value			24.996		

I: Alaxan; II: Dibulaxan; III: Febro.
